# Supplementary material for: An open-source framework for large-scale, flexible evaluation of biomedical text mining systems
Source: J Biomed Discov Collab. 2008 Jan 29;3:1. doi: 10.1186/1747-5333-3-1 (PMC2276192; doi:10.1186/1747-5333-3-1)
Supplement: Additional file 1 — Evaluation platform source code. The additional file contains the software evaluation framework discussed in this paper. The most current release can be downloaded from SourceForge.net [1]. See the accompanying README file for instructions on installation and use. Also included in the distribution is a collection of UIMA wrappers for some commonly used BioNLP tools and annotated corpora. [file 1747-5333-3-1-S1.zip › bionlp_uima_repository-1.0.1/docs/api/src-html/edu/uchsc/ccp/util/nlp/parser/corpus/BioIECorpusParser.html]

```
001    package edu.uchsc.ccp.util.nlp.parser.corpus;
002    
003    import java.io.BufferedReader;
004    import java.io.File;
005    import java.io.IOException;
006    import java.util.ArrayList;
007    import java.util.HashSet;
008    import java.util.List;
009    import java.util.Set;
010    import java.util.regex.Matcher;
011    import java.util.regex.Pattern;
012    
013    import edu.uchsc.ccp.util.nlp.GenericTextUtils;
014    import edu.uchsc.ccp.util.nlp.annotation.AnnotationSet;
015    import edu.uchsc.ccp.util.nlp.annotation.Annotator;
016    import edu.uchsc.ccp.util.nlp.annotation.TextAnnotation;
017    import edu.uchsc.ccp.util.nlp.document.GenericDocument;
018    import edu.uchsc.ccp.util.nlp.mention.ClassMentionTypes;
019    
020    /**
021     * Parser for the BioIE corpus. <br>
022     * <br>
023     * Output Classes:<br>
024     * <ul>
025     * <li>protein</li>
026     * <li>rna</li>
027     * <li>generic gene</li>
028     * </ul>
029     * 
030     * @author williamb
031     * 
032     */
033    public class BioIECorpusParser extends CorpusParser {
034        public final static int DOCUMENT_COLLECTION_ID = 11;
035        
036        private final static boolean DEBUG = false;
037    
038        private static List<TextAnnotation> allAnnotations = new ArrayList<TextAnnotation>();
039    
040        private static List<String[]> allDocuments = new ArrayList<String[]>();
041    
042        private static Set<String> typeSet = new HashSet<String>();
043    
044        private static final String GENE_PROTEIN_TYPE = "gene-protein";
045    
046        private static final String GENE_RNA_TYPE = "gene-rna";
047    
048        private static final String GENE_GENERIC_TYPE = "gene-generic";
049    
050    
051    
052        private static String documentID;
053    
054        private final static Annotator annotator = new Annotator(new Integer(12), "", "BioIE", "UPenn");
055    
056        private final static AnnotationSet annotationSet = new AnnotationSet(new Integer(10), "BioIE Oncology Gold Annotations",
057                "Annotations extracted from the BioIE oncology corpus.");
058    
059        private static int titleOffsetCount = 0;
060    
061        private static int abstractOffsetCount = 0;
062    
063        private static List<Integer> titleLineBreakIndexes = new ArrayList<Integer>();
064    
065        private static List<Integer> abstractLineBreakIndexes = new ArrayList<Integer>();
066    
067        private String[] corpusFileList;
068    
069        private int currentFileIndex;
070    
071        private File corpusDirectory;
072    
073        private static GenericDocument nextTitleDocument = null;
074    
075        private static GenericDocument nextAbstractDocument = null;
076    
077        private boolean returnedTitleDocument = false;
078    
079        private boolean returnedAbstractDocument = false;
080    
081        private static int annotationID = 0;
082    
083        /**
084         * The corpus directory must contain the source files and word-freak annotation files
085         * 
086         * @param corpusDirectory
087         */
088        public BioIECorpusParser(String corpusDirectory) {
089            File dir = new File(corpusDirectory);
090            if (dir.isDirectory()) {
091                this.corpusFileList = dir.list();
092            } else {
093                error("Input parameter: '" + corpusDirectory + "' must be a directory. Please adjust and try again");
094                System.exit(-1);
095            }
096            this.corpusDirectory = new File(corpusDirectory);
097            this.currentFileIndex = 0;
098        }
099    
100        @Override
101        public boolean hasNext() {
102            /* if we have not yet returned the abstract document, then return true */
103            if (returnedTitleDocument && !returnedAbstractDocument) {
104                return true;
105            } else {
106                /* we have already returned the abstract document, so check to see if there are new title and abstract documents to return */
107                try {
108                    return hasNextDocument();
109                } catch (IOException e) {
110                    error("Error while parsing the Bio1 corpus file.");
111                    e.printStackTrace();
112                    return false;
113                }
114            }
115        }
116    
117        @Override
118        public GenericDocument next() {
119            if (!returnedTitleDocument) {
120                returnedTitleDocument = true;
121                return nextTitleDocument;
122            } else {
123                returnedAbstractDocument = true;
124                return nextAbstractDocument;
125            }
126        }
127    
128        public void close() throws IOException {
129        }
130    
131        private boolean hasNextDocument() throws IOException {
132            /* check for completion */
133            if (currentFileIndex != corpusFileList.length) {
134                String currentFilePath = corpusDirectory.getAbsolutePath() + "/" + corpusFileList[currentFileIndex++];
135                returnedAbstractDocument = false;
136                returnedTitleDocument = false;
137                annotationID = 0;
138                File f = new File(currentFilePath);
139                /* ignore the corpus-list and tag-list files */
140                if (f.isFile()) {
141                    if (f.getName().endsWith(".src")) {
142                        /* process each file */
143    
144                        BufferedReader br = GenericTextUtils.openReader(currentFilePath);
145                        int numBlankLines = countBlankLines(br);
146                        br.close();
147                        br = GenericTextUtils.openReader(currentFilePath);
148                        String[] titleAbstractText = BioIECorpusParser.parseBioIE_SourceFile(br, numBlankLines);
149                        br.close();
150    
151                        br = GenericTextUtils.openReader(currentFilePath + ".ann");
152                        BioIECorpusParser.parseBioIE_AnnotationFile(br, documentID, titleAbstractText[0], titleAbstractText[1]);
153                        br.close();
154                        return true;
155                    } else {
156                        /* we have probably come across a .ann file, so move on to the next... */
157                        return hasNextDocument();
158                    }
159                } else {
160                    /*
161                     * do nothing.. i.e. don't recurse into any directories within the corpusDirectory, but we should check for other documents.. this
162                     * case will only occur if the user has added an extra directory
163                     */
164                    return hasNextDocument();
165                }
166            } else {
167                /* we have cycled through all of the corpus documents, so return false */
168                return false;
169            }
170        }
171    
172        public static void reset() {
173            titleLineBreakIndexes = new ArrayList<Integer>();
174            abstractLineBreakIndexes = new ArrayList<Integer>();
175            allAnnotations = new ArrayList<TextAnnotation>();
176            allDocuments = new ArrayList<String[]>();
177        }
178    
179        public static List<TextAnnotation> getTextAnnotations() {
180            return allAnnotations;
181        }
182    
183        public static List<String[]> getDocuments() {
184            return allDocuments;
185        }
186    
187        /* counts the number of blank lines before the PMID line */
188        private static int countBlankLines(BufferedReader br) throws IOException {
189            int numBlankLines = 0;
190            String line;
191            boolean stopCounting = false;
192            while ((line = br.readLine()) != null) {
193                if (line.trim().startsWith("PMID")) {
194                    stopCounting = true;
195                }
196                if (line.trim().startsWith("Publication Types:")) {
197                    stopCounting = true;
198                }
199                if (line.length() == 0 && !stopCounting) {
200                    numBlankLines++;
201                }
202            }
203            return numBlankLines;
204        }
205    
206        public static void processBioIECorpusDirectory(String directory) throws IOException {
207            allAnnotations = new ArrayList<TextAnnotation>();
208            allDocuments = new ArrayList<String[]>();
209    
210            File dir = new File(directory);
211            if (dir.isDirectory()) {
212    
213                String[] files = dir.list();
214    
215                for (String file : files) {
216                    File f = new File(dir.getAbsolutePath() + "/" + file);
217                    /* ignore the corpus-list and tag-list files */
218                    if (f.isFile()) {
219                        if (f.getName().endsWith(".src")) {
220                            /* process each file */
221    
222                            BufferedReader br = GenericTextUtils.openReader(dir.getAbsolutePath() + "/" + file);
223                            int numBlankLines = countBlankLines(br);
224                            br.close();
225                            br = GenericTextUtils.openReader(dir.getAbsolutePath() + "/" + file);
226                            String[] titleAbstractText = BioIECorpusParser.parseBioIE_SourceFile(br, numBlankLines);
227                            br.close();
228    
229                            br = GenericTextUtils.openReader(dir.getAbsolutePath() + "/" + file + ".ann");
230                            BioIECorpusParser.parseBioIE_AnnotationFile(br, documentID, titleAbstractText[0], titleAbstractText[1]);
231                            br.close();
232                        }
233                    }
234                }
235    
236                /* print out all types */
237                for (String type : typeSet) {
238                    System.out.println("TYPE: " + type);
239                }
240    
241                // BioIEParser.storeOutput();
242    
243            } else {
244                System.err.println("ERROR -- TexasCorpusParser: Input is not a directory.");
245            }
246        }
247    
248        /**
249         * output a list of the pmids in the BioIE collection
250         * 
251         */
252        public static Set<String> getListOfBioIEPMIDs(String directory) throws IOException {
253            Set<String> pmids = new HashSet<String>();
254    
255            File dir = new File(directory);
256            if (dir.isDirectory()) {
257    
258                String[] files = dir.list();
259    
260                for (String file : files) {
261                    File f = new File(dir.getAbsolutePath() + "/" + file);
262                    /* ignore the corpus-list and tag-list files */
263                    if (f.isFile()) {
264                        if (f.getName().endsWith(".src")) {
265                            /* process each file */
266    
267                            BufferedReader br = GenericTextUtils.openReader(dir.getAbsolutePath() + "/" + file);
268                            int numBlankLines = countBlankLines(br);
269                            br.close();
270                            br = GenericTextUtils.openReader(dir.getAbsolutePath() + "/" + file);
271                            String[] titleAbstractText = BioIECorpusParser.parseBioIE_SourceFile(br, numBlankLines);
272                            String pmid = titleAbstractText[2];
273                            if (!pmids.contains(pmid)) {
274                                pmids.add(pmid);
275                            } else {
276                                System.err.println("Duplicate pmid found in BioIE corpus: " + pmid);
277                            }
278                            br.close();
279                        }
280                    }
281                }
282            }
283    
284            System.out.println("Returning: " + pmids.size() + " pmids.");
285    
286            return pmids;
287        }
288    
289        /**
290         * returns a String Array of size 2<br>
291         * String[0] = title<br>
292         * String[1] = abstract<br>
293         * String[2] = documentID<br>
294         * 
295         * @param br
296         * @return
297         * @throws IOException
298         */
299        public static String[] parseBioIE_SourceFile(BufferedReader br, int numBlankLines) throws IOException {
300    
301            int blankLineCount = 0;
302            titleOffsetCount = 0;
303            abstractOffsetCount = 0;
304            int byteCount = 0;
305    
306            String titleText = "";
307            String abstractText = "";
308    
309            titleLineBreakIndexes = new ArrayList<Integer>();
310            abstractLineBreakIndexes = new ArrayList<Integer>();
311    
312            /*
313             * some of the files have slightly different formats; some have 2 "Comment in" lines, some "Erratum", others do not have an Institution line.
314             * The input numBlankLines is used to distinguish between formats. If numBlankLines = 4 then this file has no Institution line.
315             */
316    
317            // int titleBlankLineCount = 1;
318            // int abstractBlankLineCount = 4;
319            // if (numBlankLines == 4) {
320            // abstractBlankLineCount = 3;
321            // }
322            boolean foundTitle = false;
323            boolean foundAbstract = false;
324            boolean oneMoreLine = false;
325            boolean seenBlankLineAfterTitle = false;
326    
327            String line;
328            while ((line = br.readLine()) != null) {
329                byteCount += (line.length() + 2);
330                if (line.startsWith("PMID")) {
331                    Pattern idPattern = Pattern.compile("PMID:\\s(\\d+)\\s");
332                    Matcher m = idPattern.matcher(line);
333                    if (m.find()) {
334                        documentID = m.group(1);
335                    } else {
336                        System.err.println("ERROR -- BioIEParser: Expected PMID, but did not match.");
337                    }
338                } else if (line.length() == 0) {
339                    blankLineCount++;
340                    if (!foundTitle) {
341                        titleOffsetCount += 2;
342                    } else {
343                        seenBlankLineAfterTitle = true;
344                    }
345                    if (!foundAbstract) {
346                        abstractOffsetCount += 2;
347                    }
348                    oneMoreLine = false;
349                } else if (line.trim().startsWith("Comment in:")) {
350                    titleOffsetCount += line.length() + 2;
351                    abstractOffsetCount += line.length() + 2;
352                    oneMoreLine = true;
353                } else if (line.trim().startsWith("Erratum in:")) {
354                    titleOffsetCount += line.length() + 2;
355                    abstractOffsetCount += line.length() + 2;
356                    oneMoreLine = true;
357                } else if (blankLineCount + 1 == numBlankLines) {
358                    /* then we are adding the line to the abstract */
359                    foundAbstract = true;
360                    abstractText += (line + " ");
361                    abstractLineBreakIndexes.add(byteCount - 2);
362                } else if (blankLineCount > 0 && !foundAbstract && !oneMoreLine && !seenBlankLineAfterTitle) {
363                    /* then we are adding the line to the title */
364                    foundTitle = true;
365                    titleText += (line + " ");
366                    titleLineBreakIndexes.add(byteCount - 2); /*
367                                                                 * -2 because entities at the beginning of lines were getting chopped by a single
368                                                                 * character
369                                                                 */
370                    abstractOffsetCount += line.length() + 2;
371                } else {
372                    if (!foundAbstract) {
373                        abstractOffsetCount += line.length() + 2;
374                    }
375                    if (!foundTitle) {
376                        titleOffsetCount += line.length() + 2;
377                    }
378    
379                }
380            }
381    
382            if (DEBUG) {
383                System.err.println("DEBUG -- BioIEParser: TitleOffset: " + titleOffsetCount + "  AbstractOffset: " + abstractOffsetCount);
384                System.err.print("DEBUG -- BioIEParser: Abstract Line Break Indexes: ");
385                for (Integer i : abstractLineBreakIndexes) {
386                    System.err.print(i + ", ");
387                }
388                System.err.println(";");
389            }
390    
391            String[] t = new String[2];
392            t[0] = documentID + "-t";
393            t[1] = titleText.trim();
394            allDocuments.add(t);
395    
396            /* create the nextTitleDocument here */
397            nextTitleDocument = new GenericDocument();
398            nextTitleDocument.setDocumentID(documentID + "-t");
399            nextTitleDocument.setDocumentText(titleText.trim());
400    
401            String[] a = new String[2];
402            a[0] = documentID + "-a";
403            a[1] = abstractText.trim();
404            allDocuments.add(a);
405    
406            nextAbstractDocument = new GenericDocument();
407            nextAbstractDocument.setDocumentID(documentID + "-a");
408            nextAbstractDocument.setDocumentText(abstractText.trim());
409    
410            String[] titleAbstractText = new String[3];
411            titleAbstractText[0] = titleText;
412            titleAbstractText[1] = abstractText;
413            titleAbstractText[2] = documentID;
414            return titleAbstractText;
415        }
416    
417        public static void parseBioIE_AnnotationFile(BufferedReader br, String documentID, String titleText, String abstractText)
418                throws IOException {
419    
420            Pattern annotPattern = Pattern.compile("<Annotation type=\"(.*?)\" span=\"(\\d+)\\.\\.(\\d+)\"");
421            Matcher m;
422    
423            String line;
424            while ((line = br.readLine()) != null) {
425    
426                if (line.trim().startsWith("<Annotation type=")) {
427                    m = annotPattern.matcher(line);
428                    if (m.find()) {
429                        String type = m.group(1);
430                        typeSet.add(type);
431    
432                        if (type.equals(GENE_GENERIC_TYPE) || type.equals(GENE_PROTEIN_TYPE) || type.equals(GENE_RNA_TYPE)) {
433    
434                            int spanStart = Integer.parseInt(m.group(2));
435                            int spanEnd = Integer.parseInt(m.group(3));
436    
437                            if (DEBUG) {
438                                System.err.println("ABOUT TO CREATE ANNOTATION, ORIGINAL SPAN: docID: " + documentID + "[" + spanStart + ".."
439                                        + spanEnd + "]");
440                            }
441    
442                            determineSectionAndCreateAnnotation(documentID, type, spanStart, spanEnd, titleText, abstractText);
443                        }
444                    } else {
445                        System.err.println("ERROR -- BioIEParser: Expected Annotation, but did not match.");
446                    }
447                }
448            }
449        }
450    
451        private static void determineSectionAndCreateAnnotation(String documentID, String type, int spanStart, int spanEnd, String titleText,
452                String abstractText) {
453    
454            String coveredText = null;
455            int finalLineBreakOfTitle = titleLineBreakIndexes.get(titleLineBreakIndexes.size() - 1);
456            boolean inTitle = false;
457            boolean inAbstract = false;
458            if (spanStart < finalLineBreakOfTitle) {
459                /* then this annotation is in the title */
460                int spanStartOffsetDueToLineBreaksInTitle = 0;
461                int spanEndOffsetDueToLineBreaksInTitle = 0;
462                for (Integer index : titleLineBreakIndexes) {
463                    if (spanStart > index) {
464                        spanStartOffsetDueToLineBreaksInTitle++;
465                    }
466                    if (spanEnd > index) {
467                        spanEndOffsetDueToLineBreaksInTitle++;
468                    }
469                }
470    
471                spanStart = spanStart - spanStartOffsetDueToLineBreaksInTitle - titleOffsetCount;
472                spanEnd = spanEnd - spanEndOffsetDueToLineBreaksInTitle - titleOffsetCount;
473    
474                coveredText = titleText.substring(spanStart, spanEnd);
475                documentID += "-t";
476                inTitle = true;
477            } else {
478                /* this annotation is in the abstract */
479                int spanStartOffsetDueToLineBreaksInAbstract = 0;
480                int spanEndOffsetDueToLineBreaksInAbstract = 0;
481                for (Integer index : abstractLineBreakIndexes) {
482                    if (spanStart > index) {
483                        spanStartOffsetDueToLineBreaksInAbstract++;
484                    }
485                    if (spanEnd > index) {
486                        spanEndOffsetDueToLineBreaksInAbstract++;
487                    }
488                }
489    
490                spanStart = spanStart - spanStartOffsetDueToLineBreaksInAbstract - abstractOffsetCount;
491                spanEnd = spanEnd - spanEndOffsetDueToLineBreaksInAbstract - abstractOffsetCount;
492    
493                try {
494                    coveredText = abstractText.substring(spanStart, spanEnd);
495                } catch (StringIndexOutOfBoundsException siobe) {
496                    System.err.println("ERROR -- StringIndexOutOfBoundsException in document: " + documentID);
497                }
498                documentID += "-a";
499                inAbstract = true;
500            }
501    
502            // /* 11290569, 9207791 - examples where the coveredtext includes a line break */
503            // String coveredText = documentText.substring(spanStart, spanEnd).replaceAll("\u0009","").replaceAll("\\n\\s?"," ");
504    
505            TextAnnotation ta = null;
506            if (type.equals(GENE_PROTEIN_TYPE)) {
507                ta = createTextAnnotation(documentID, DOCUMENT_COLLECTION_ID, spanStart, spanEnd, ClassMentionTypes.PROTEIN,
508                        coveredText, annotator, annotationSet, annotationID++);
509                
510            } else if (type.equals(GENE_RNA_TYPE)) {
511                ta = createTextAnnotation(documentID, DOCUMENT_COLLECTION_ID, spanStart, spanEnd, ClassMentionTypes.RNA,
512                        coveredText, annotator, annotationSet, annotationID++);
513            } else if (type.equals(GENE_GENERIC_TYPE)) {
514                ta = createTextAnnotation(documentID, DOCUMENT_COLLECTION_ID, spanStart, spanEnd, ClassMentionTypes.GENE_GENERIC,
515                        coveredText, annotator, annotationSet, annotationID++);
516            } else {
517                System.err.println("Unexpected annotation type discovered in BioIE corpus: " + type);
518            }
519    
520            allAnnotations.add(ta);
521            if (inTitle) {
522                nextTitleDocument.addAnnotation(ta);
523            } else if (inAbstract) {
524                nextAbstractDocument.addAnnotation(ta);
525            } else {
526                System.err.println("WARNING -- BioIEParser: Encounted annotation not in title or abstract.");
527            }
528        }
529    
530        // private static TextAnnotation createTextAnnotation(String documentID, int documentCollectionID, int spanStart, int spanEnd,
531        // String classMentionType, String coveredText, Annotator annotator, AnnotationSet annotationSet) {
532        // TextAnnotation ta = new TextAnnotation();
533        //
534        // /* set the annotator */
535        // ta.setAnnotator(annotator);
536        //
537        // /* add the annotation set */
538        // ta.addAnnotationSet(annotationSet);
539        //
540        // /* set the documentID */
541        // ta.setDocumentID(documentID);
542        //
543        // /* set the documentCollectionID */
544        // ta.setDocumentCollectionID(documentCollectionID);
545        //
546        // /* set the span */
547        // ta.setAnnotationSpanEnd(spanEnd);
548        // ta.setAnnotationSpanStart(spanStart);
549        //
550        // /* set the covered text */
551        // ta.setCoveredText(coveredText);
552        //
553        // /* set the class mention */
554        // ClassMention cm = new ClassMention();
555        // cm.setMentionName(classMentionType);
556        // ta.setClassMention(cm);
557        //
558        // return ta;
559        // }
560    
561    //    public static void storeOutput() throws IOException {
562    //        /* now store results in the database */
563    //        /* now add documents to the database */
564    //        for (String[] idTextPair : allDocuments) {
565    //            String documentID = idTextPair[0];
566    //            String documentText = idTextPair[1];
567    //
568    //            documentText = documentText.replaceAll("'", "''");
569    //
570    //            char TorA = documentID.charAt(documentID.length() - 1);
571    //
572    //            String sqlStr = "INSERT INTO BIOIE_ONCOLOGY_CORPUS VALUES ('" + documentID + "', '" + Character.toUpperCase(TorA) + "', '"
573    //                    + documentText + "')";
574    //            System.out.println("SQLSTR: " + sqlStr);
575    //            Database_Util.execute(AnnotationDBProperties.CONNECTION_PROPERTIES_FILE, sqlStr);
576    //        }
577    //
578    //        String sqlldrFileName = "bioieOnc_Annotations.sqlldr";
579    //        String runLoadFileName = "loadBioIEOncAnnotations.sh";
580    //
581    //        /* add the annotations to the database in the form of an sqlldr file */
582    //        /* create the sqlldr input file */
583    //        PrintStream sqlldrFileStream = GenericTextUtils.openPrintStream(AnnotationDBProperties.EXTERNAL_BASE_ANNOTATION_DIR
584    //                + "/inputFiles/" + sqlldrFileName);
585    //        AnnotationDB_Util.printTextAnnotationListToSqlldrFile(sqlldrFileStream, allAnnotations, new ArrayList<String>(), true,
586    //                AnnotationDBProperties.CONNECTION_PROPERTIES_FILE, false);
587    //        sqlldrFileStream.close();
588    //
589    //        /* add the parsed gene annotations to the database */
590    //        AnnotationDB_Util.loadAnnotationsIntoDB(AnnotationDBProperties.EXTERNAL_BASE_ANNOTATION_DIR,
591    //                AnnotationDBProperties.LOCAL_BASE_ANNOTATION_DIR, sqlldrFileName, AnnotationDBProperties.SQLLDR_CONTROL_FILE_TEMPLATE,
592    //                runLoadFileName, AnnotationDBProperties.ANNOTATION_DB_STATUS_FLAG_FILE, true);
593    //
594    //    }
595    
596        /**
597         * args[0] = BioIE corpus directory (where wordfreak files reside)
598         * 
599         * @param args
600         */
601        public static void main(String[] args) {
602    
603            try {
604                // BioIEParser.processBioIECorpusDirectory(args[0]);
605    
606                Set<String> pmids = BioIECorpusParser.getListOfBioIEPMIDs(args[0]);
607                for (String pmid : pmids) {
608                    System.out.println(pmid);
609                }
610    
611            } catch (IOException e) {
612                e.printStackTrace();
613            }
614        }
615    
616    }
```
